# Climatic, land-use and socio-economic factors can predict malaria dynamics at fine spatial scales relevant to local health actors: Evidence from rural Madagascar

**DOI:** 10.1371/journal.pgph.0001607

**Published:** 2023-02-22

**Authors:** Julie D. Pourtois, Krti Tallam, Isabel Jones, Elizabeth Hyde, Andrew J. Chamberlin, Michelle V. Evans, Felana A. Ihantamalala, Laura F. Cordier, Bénédicte R. Razafinjato, Rado J. L. Rakotonanahary, Andritiana Tsirinomen’ny Aina, Patrick Soloniaina, Sahondraritera H. Raholiarimanana, Celestin Razafinjato, Matthew H. Bonds, Giulio A. De Leo, Susanne H. Sokolow, Andres Garchitorena

**Affiliations:** 1 Biology Department, Stanford University, Stanford, CA, United States of America; 2 Hopkins Marine Station, Stanford University, Pacific Grove, CA, United States of America; 3 School of Medicine, Stanford University, Stanford, CA, United States of America; 4 MIVEGEC, Université de Montpellier, CNRS, IRD, Montpellier, France; 5 Department of Global Health and Social Medicine, Harvard Medical School, Boston, MA, United States of America; 6 NGO Pivot, Ifanadiana, Madagascar; 7 Programme National de Lutte contre le Paludisme, Ministère de la Santé Publique, Antananarivo, Madagascar; 8 Woods Institute for the Environment, Stanford University, Stanford, CA, United States of America; 9 Marine Science Institute and Department of Ecology, Evolution and Marine Biology, University of California, Santa Barbara, CA, United States of America; University of Oslo Faculty of Medicine: Universitetet i Oslo Det medisinske fakultet, NORWAY

## Abstract

While much progress has been achieved over the last decades, malaria surveillance and control remain a challenge in countries with limited health care access and resources. High-resolution predictions of malaria incidence using routine surveillance data could represent a powerful tool to health practitioners by targeting malaria control activities where and when they are most needed. Here, we investigate the predictors of spatio-temporal malaria dynamics in rural Madagascar, estimated from facility-based passive surveillance data. Specifically, this study integrates climate, land-use, and representative household survey data to explain and predict malaria dynamics at a high spatial resolution (i.e., by Fokontany, a cluster of villages) relevant to health care practitioners. Combining generalized linear mixed models (GLMM) and path analyses, we found that socio-economic, land use and climatic variables are all important predictors of monthly malaria incidence at fine spatial scales, via both direct and indirect effects. In addition, out-of-sample predictions from our model were able to identify 58% of the Fokontany in the top quintile for malaria incidence and account for 77% of the variation in the Fokontany incidence rank. These results suggest that it is possible to build a predictive framework using environmental and social predictors that can be complementary to standard surveillance systems and help inform control strategies by field actors at local scales.

## Introduction

Interventions for malaria control have been highly successful in many countries over the last few decades, leading to a 47% decrease in mortality rates globally between 2001 and 2013 [1]. However, access to preventative and curative health care remains limited in many rural areas of low-income countries, where the burden of malaria is concentrated [2]. This problem is expected to intensify as population growth and climate change are projected to increase the number of people at risk for malaria in these areas [1]. Ensuring universal access to malaria diagnostics and treatment thus remains a key goal in the global malaria strategic plan for 2016–2030, which recognizes that improved surveillance is essential to inform those efforts [1]. However, limited human and financial resources are significant obstacles to reaching this goal in low-income-countries [3]. Most malaria surveillance occurs passively, only capturing those cases that reach health facilities, missing the vast majority of infections where access to health care is low [4–6]. To help overcome these obstacles, improved methods to predict malaria incidence at local scales could help health care practitioners optimize the distribution of limited resources when and where they are most needed.

There has been substantial progress on predicting malaria at regional and national scales: aggregations of data from national Demographic and Health Surveys or from national surveillance systems, in combination with large-scale remotely sensed environmental data available in public repositories, are able to explain and predict spatial patterns in malaria at the global and regional scale with relatively good resolution. A leading example is the Malaria Atlas Project, which maps malaria incidence and relevant variables across the world [7], but many other studies are done at these scales [8–12]. While global and regional patterns may be useful for international organizations or national governments to estimate total malaria burdens and medical treatment needs [12], they cannot necessarily inform malaria control at the local level (e.g. within a government district), where most control activities are actually implemented [13]. Malaria incidence can be highly heterogeneous at small spatial scales [14], so a key need to improve local strategies to curb malaria transmission is to better understand how disease risk varies at fine spatial and temporal scales, and whether there are predictable factors (environmental, socio-economic, climatic) driving this variability. This could help medical practitioners and local health programs anticipate resource needs or inform the implementation of targeted control activities. There remain, however, substantial challenges to downscaling malaria predictions at these scales, especially the resolution and quality of current routine surveillance systems and the lower heterogeneity in environmental and socio-demographic predictors at small scales.

High quality data on malaria incidence is limited in many countries. Studies attempting to predict malaria patterns often rely on surveys or active surveillance methods, which have limited geographic and temporal scope [14–17]. Routine health system data provide a more sustainable data source and are available in most countries, but the spatial resolution of such data is low when aggregate numbers of cases over a catchment area are reported, as is often the case, rather than the precise locations of cases [18]. In addition, the quality of health facility-based data can be highly variable and difficult to assess [19], and there are many geographic and financial barriers to care, resulting in surveillance data biased towards populations living close to higher-quality health centers [18]. The health system burden may thus not be an accurate representation of the community burden [19]. As a result, new ways of reporting data and adjusting it to remove known biases are necessary before one can even attempt to understand local drivers of malaria, and predict the spatio-temporal variability of incidence.

Many social and environmental factors have been shown to influence exposure and vulnerability to malaria infection. Household wealth is generally associated with preventive behaviours, while the coverage and use of insecticide-treated bed nets are effective in reducing malaria transmission [20], both of which can be derived from national surveys. Climatic and environmental factors such as temperature, precipitation and land use have been shown to affect mosquito habitat and life history traits [21,22]. The resolution of many of these predictors is constantly improving with the help of remote sensing, as is the resolution of predictions [23]. At national or regional scales, the heterogeneity (range of variation) of these predictors is high, making it feasible to detect associations of climatic variables with malaria incidence or prevalence data [9,17,24]. However, measuring heterogeneity presents a challenge at finer scales, even with environmental data remotely sensed at higher spatial resolution: a smaller range of variation in predictors, an increase in spatial auto-correlation, and a greater role of additional unobservable (stochastic) processes and behavioural factors can make fine scale projections of spatial variability incredibly challenging. As a result, it is unclear whether more localized studies, even with the best available data, can find consistent predictors of local disease dynamics at the finer scales at which health care interventions occur [25].

The aim of this work is to explore whether data gathered from routine surveillance systems can be used along with ecological, environmental and socioeconomic information to improve our understanding of spatio-temporal malaria dynamics at fine scales. We built on previous work where passive surveillance data on malaria incidence from the rural district of Ifanadiana, in south-eastern Madagascar, was adjusted to correct for reporting biases derived from financial and geographic barriers to health care [18]. The resulting data provide adjusted monthly malaria incidence per Fokontany (i.e. cluster of villages), improving nearly ten-fold the spatial resolution of malaria incidence rates as compared with aggregate health facility reports. We coupled this with satellite information on climate and land-use dynamics, as well as socio-economic information from a longitudinal cohort study representative of the district population. We then identified correlates of malaria incidence across time and space with a mixed-effects generalized linear model and explored causal relationships among these variables using structural equation modelling. Ifanadiana was an ideal region to explore these questions, as heterogeneity in climate, land-use, and household wealth is high relative to its spatial size [25]. Improving predictions of malaria dynamics at a high spatial resolution could pave the way towards local forecasting and early warning systems with operational applications for on-the-ground distribution of health care resources.

## Results

**Fig 1:**
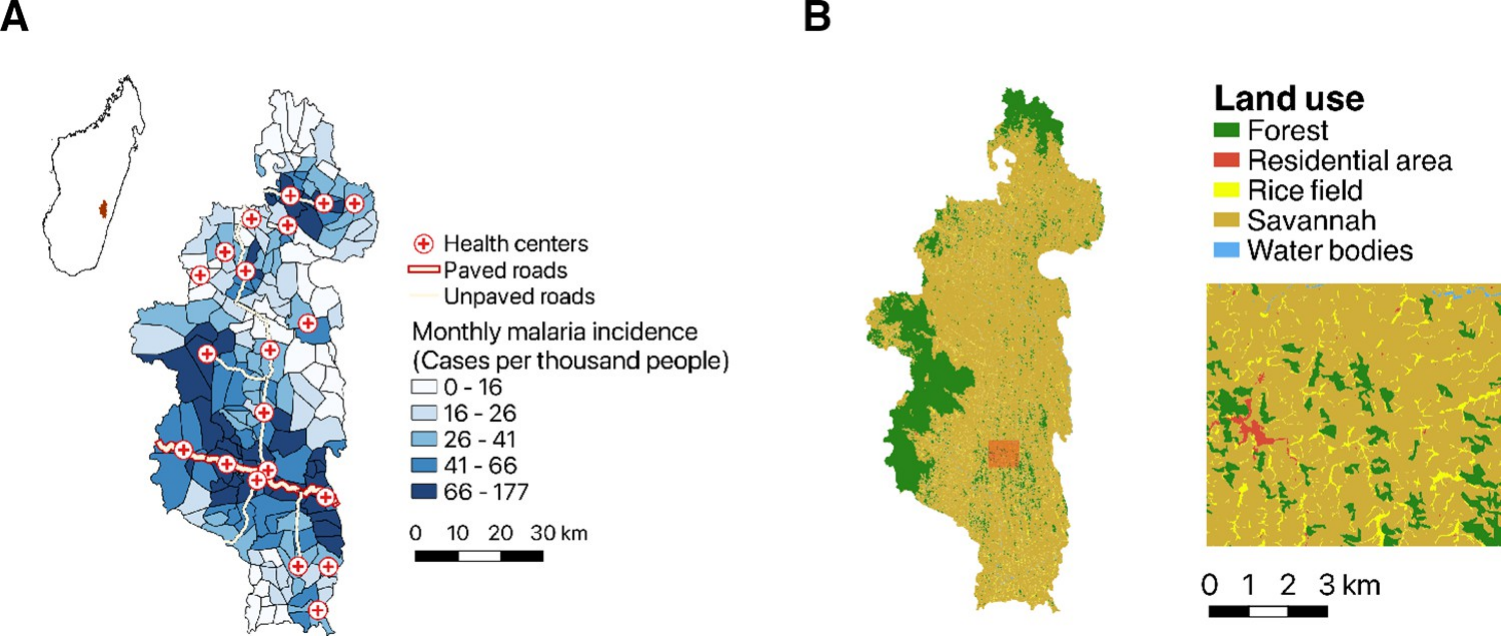
(A) Map of roads (paved and unpaved), health centers and adjusted monthly malaria incidence averaged over four years in Ifanadiana, and its location in Madagascar (inset map). (B) Broad and detailed view of the land use map and its five land classes. Both maps were made with QGIS, with boundary data from OCHA (https://data.humdata.org/dataset/cod-ab-mdg) under a CC BY 4.0 License and land use data available through OpenStreetMap.

A total of 326,334 malaria cases were estimated in the adjusted dataset for Ifanadiana from January 2014 to December 2017, equivalent to an average of 1.65 infections per capita over that four-year period. We observed large variations in infections across space and time. Total infections per capita over four years ranged from 0 to 6.9 across Fokontany. This corresponds to an average incidence of 42 cases per thousand people (‰) per month, ranging from 0 to 177 ‰ across Fokontany (Fig 1A). The discrepancy between the expected total number of infections based on these averages and the observed total number of infections presented above can be explained by missing data (each Fokontany was missing 4.7 months of data on average). The adjusted malaria incidence remained clustered along the main roads and in Fokontany close to health centers (Fig 1A). Malaria incidence was seasonal, with lower incidence from June to October (7–22 ‰ per month, averaged over all Fokontany and over 4 years) and higher incidence from November to May (36–92 ‰ per month, averaged over all Fokontany and over 4 years) (S1 Fig). November to May were also associated with high precipitation and high temperatures (S1 Fig). We focus our statistical analyses of malaria predictors on the high season to reduce the number of excess zeroes in our dataset and to increase our ability to capture spatial variation.

The hottest month of the year was November, with a mean land surface temperature (LST) across 4 years of 33°C (S1 Fig). The coldest month of the high season (November to May) was May, with a mean LST across 4 years of 23°C. May was also the driest month of the high season, with an average total precipitation of 65 mm. The wettest months were January and March, with a mean precipitation over four years of 330 and 384 mm respectively. Land use varied spatially, with forested areas concentrated in the West and North, and an open landscape with residential areas and rice fields in the rest of the district (Fig 1B). Spatial distributions for all predictor variables are available in S2 Fig.

### Socio-economic, land use and climatic variables accurately predicted spatial and temporal hotspots in GLMMs

**Fig 2:**
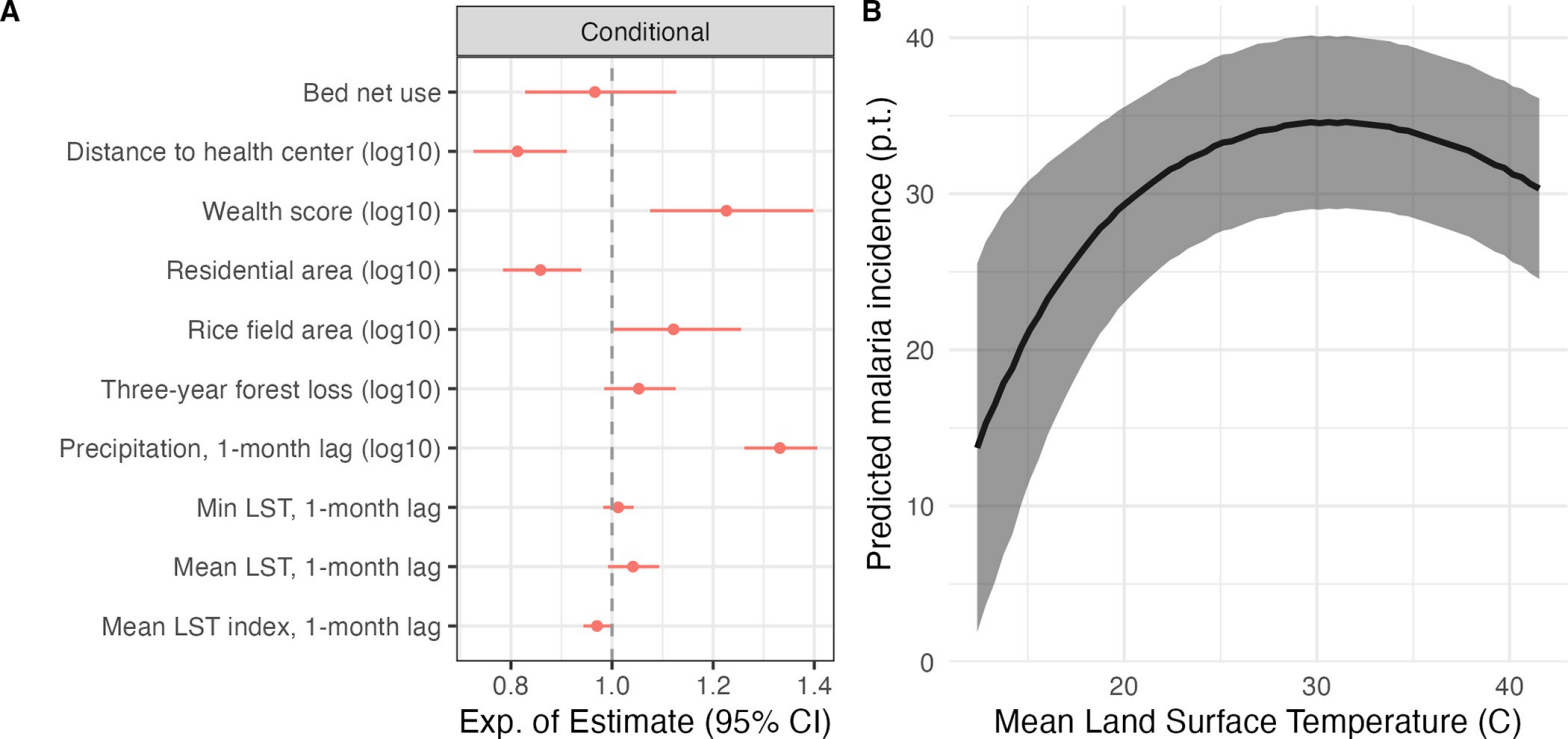
GLMM analysis. (A) Exponential of the coefficient estimates and 95% confidence interval for the conditional part of the GLMM with zero-inflated negative binomial distribution and spatial and temporal covariance structure. The exponential of the coefficient represents the expected multiplicative change in malaria incidence for a one-unit change of the predictor variable. Variables were scaled so that a one-unit change represents a change of 1 standard deviation for all variables. (B) Marginal effect of mean LST and mean LST index (one-month lag, standardized) on malaria incidence, conditioned on both conditional and zero-inflated parts of the model for a random Fokontany and random time point, with standard error interval.

The generalized linear mixed model (GLMM) included a zero-inflated negative binomial structure, fixed effects for socio-economic, land use and climatic variables (Fig 2A), and a Ornstein–Uhlenbeck and Matern covariance structure to account for temporal and spatial autocorrelation respectively (see Methods section). Overall, the conditional part of the model had a larger influence on the relationship between our predictors and malaria incidence than the zero-inflated part of the model (S1 Table). The socio-economic variables included in the GLMM were distance to health centers, wealth score and bed net use. An increase of one standard deviation (SD) in the log-transformed distance to health centers increased the odds of seeing a structural zero by 120% and decreased the expected malaria incidence by 19% (95% CI: 0.73–0.91, Fig 2A). An increase in log-transformed wealth score was associated with a 23% increase in the expected malaria incidence (95% CI: 1.08–1.40). Bed net use was not associated with a significant change in malaria incidence (95% CI: 0.83–1.13). Among land use variables, the log-transformed proportion of residential land use was negatively associated with malaria incidence, with a 14% decrease in malaria incidence with each SD change (95% CI: 0.78–0.94). The log-transformed proportion of rice fields was positively associated with malaria incidence, with a 12% increase in malaria incidence with each SD increase (95% CI:1.00–1.26). Forest loss over the previous 3 years did not significantly affect malaria incidence (95% CI:0.98–1.13). With regard to climate, a 1 SD increase in log-transformed total precipitation, lagged by one month, was associated with a 33% increase in malaria incidence (95% CI:1.26–1.41). Mean land surface temperature (LST) was associated with a 46% decrease in the odds of a structural zero (95% CI: 0.34–0.86). Finally, mean temperatures closer to the optimal temperature of 25°C for malaria (i.e., mean LST index) were associated with higher malaria incidence (cond. factor: 0.97, z.i. factor: 1.31). This resulted in a unimodal relationship between mean LST and malaria incidence (Fig 2B).

**Fig 3:**
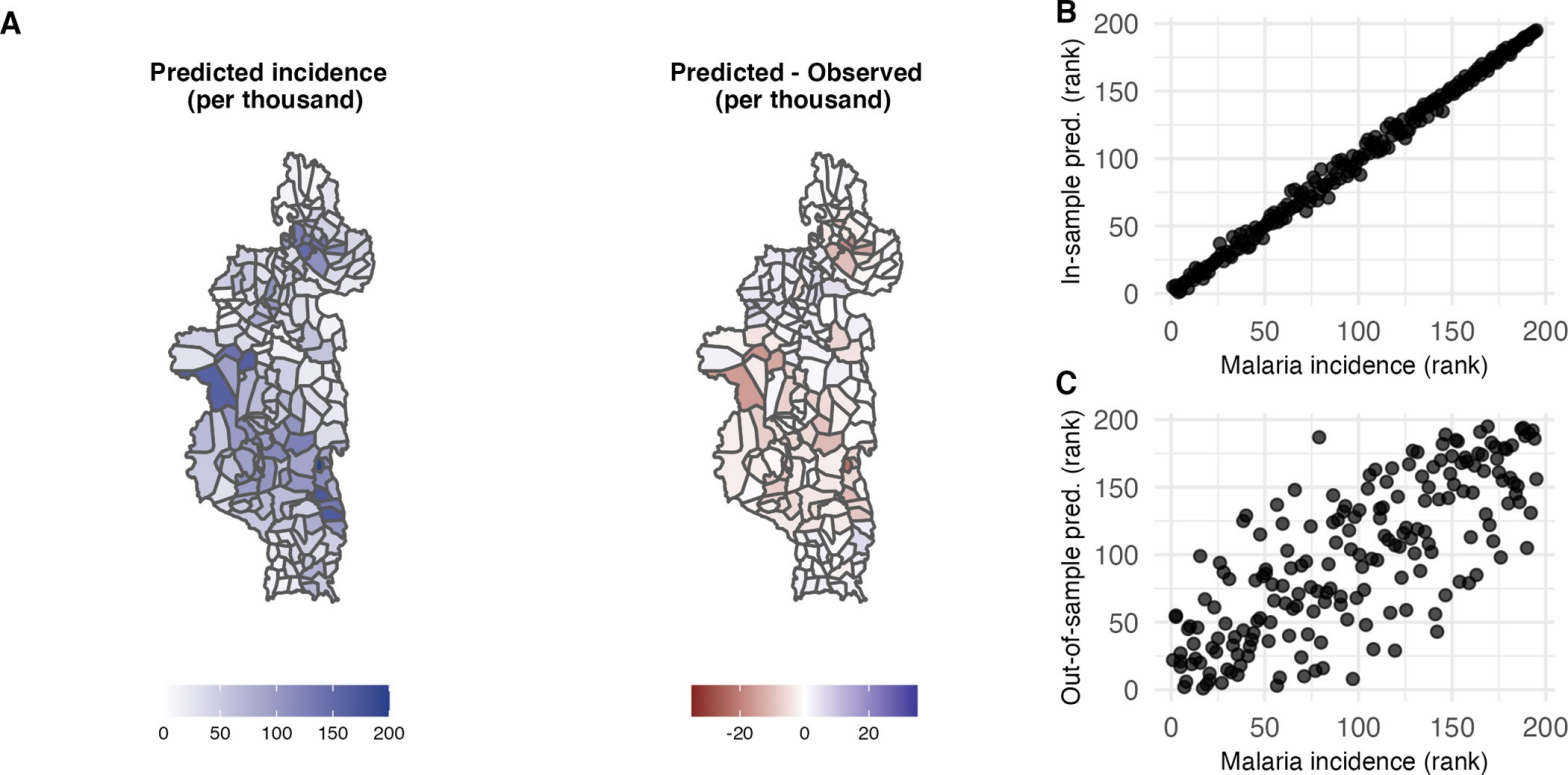
Predictions from GLMM with month and Fokontany ID as random effects. (A) Left: Within-sample predictions for monthly malaria incidence. Right: Difference between observed and predicted malaria incidence across space. Overpredictions are shown in blue and underpredictions in red. Fokontany boundary data is available from OCHA (https://data.humdata.org/dataset/cod-ab-mdg) under a CC BY 4.0 License. (B) Scatterplot of ranked within-sample malaria predictions across ranked observed malaria incidence, for data averaged across space. One point represents one Fokontany. (C) Scatterplot of ranked out-of-sample malaria predictions across ranked observed malaria incidence, for data averaged across space. One point represents one Fokontany. The model was trained on the malaria seasons from 2014 to 2016 and tested on the malaria season from November 2016 to May 2017.

We used a mixed-effect GLM with month and Fokontany ID as random effects to make predictions that are not dependent on malaria incidence in the prior month (unlike the model using a temporal covariance structure). Our in-sample model predictions across space and time had a root mean square error of 52 cases per thousand people, and a high correlation with observed malaria incidence in log-space (Pearson’s R = 0.73). When averaged over time, our predictions and observed malaria incidence were strongly correlated (Pearson’s R = 0.997, Spearman’s *ρ* = 0.997, Fig 3A and 3B). More specifically, our predictions correctly identified 39 out of 40 (top 20%) fokontany with the highest average malaria incidence from 2014 to 2017. Our model was also able to capture the seasonality of malaria incidence during the high transmission season, capturing the incidence peak from January–March for all three years (S3 Fig). When trained on the 2014–2015 and 2015–2016 malaria seasons (November-May) and tested on the 2016–2017 season, we obtained a RMSE of 56 cases per thousand people. For comparison, the mean malaria incidence per month during the high season was 60.5, ranging from 0 to 844 cases per thousand people per month. In addition, the model identified 9 out of 20 (top 10%) and 23 out of 40 (top 20%) Fokontany with the highest average malaria incidence for the 2017–2018 season, compared to an expected 2 out of 20 and 8 out of 40 if identified at random. We found a high correlation between our predictions and observed malaria incidence averaged over time (Pearson’s R = 0.69, Spearman’s *ρ* = 0.77, Fig 3C). Finally, we evaluated the performance of our model with fixed effects only. Without random effects, our model evaluated across time and space had a RMSE of 67, and we found a Pearson’s correlation coefficient of 0.50 and a Spearman rank correlation coefficient of 0.60 between our predictions and observed malaria incidence averaged across time. We obtained a RMSE of 59 for out-of-sample predictions for the 2016–2017 season.

### Path analysis provides more insight at the spatial level

**Fig 4:**
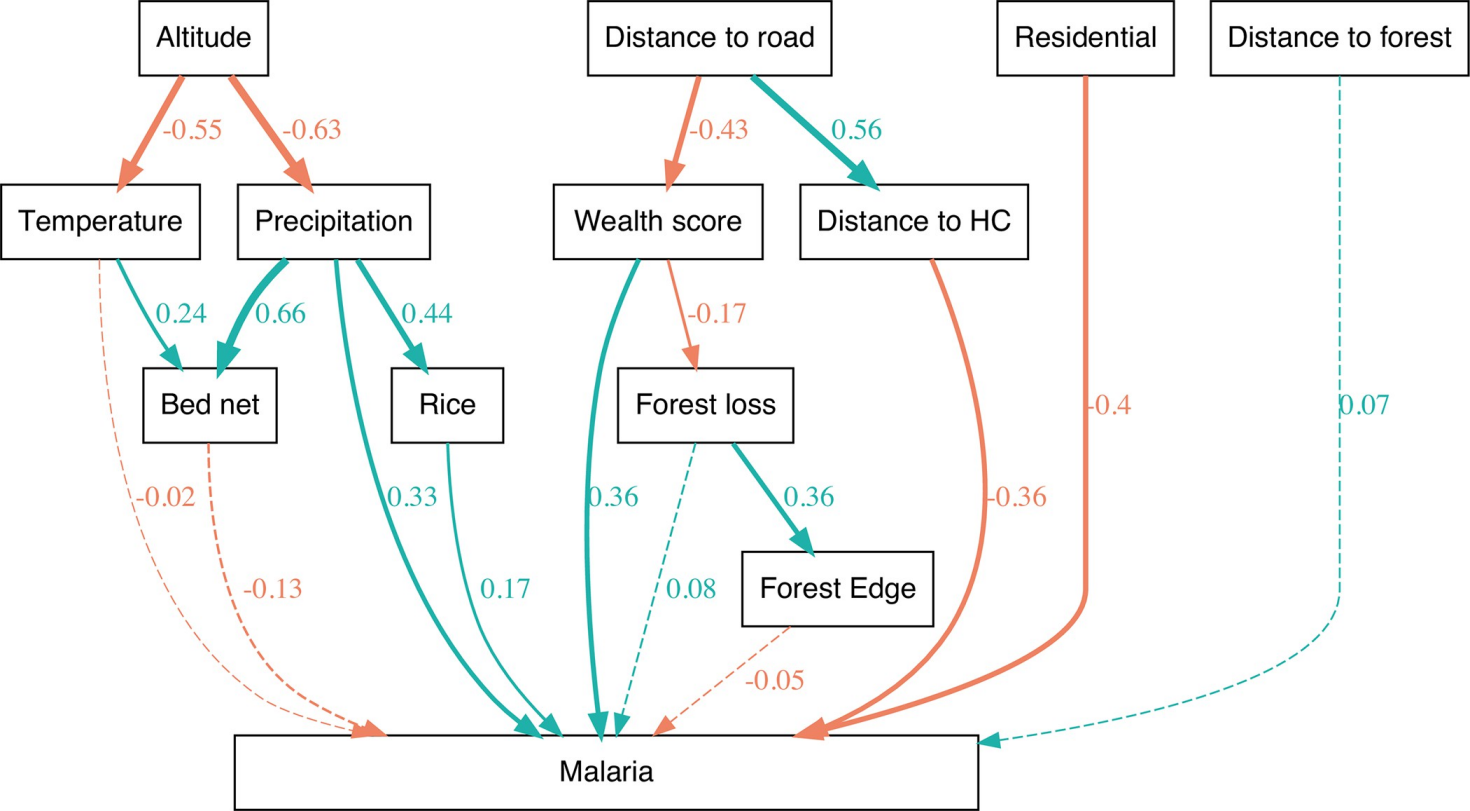
Model structure and standardized coefficient estimates for the path analysis. Positive and negative effects are shown with green and red arrows respectively. Arrow width is also proportional to coefficient estimates. Dashed arrows show non-significant effects.

We used a path analysis to further investigate the spatial relationship between our predictors and their effect on malaria incidence (Fig 4). For this, data on both malaria and its predictors were averaged by Fokontany over the study period.

Many of the relationships that were found to be significant in the path analysis confirmed our hypotheses (see Methods section). More specifically, we found that distance to the road was negatively correlated with wealth. Wealth was also associated with a decrease in forest loss, which was itself a predictor of forest edge. Altitude was associated with lower precipitation and land surface temperature. Both land surface temperature and precipitation were positively associated with bed net use. Precipitation also had a positive effect on rice field area. Direct effects between predictors and malaria incidence were mostly consistent with our GLMM results, but fewer predictors had a significant relationship with malaria: the effect of bed nets, temperature, and forest loss on malaria incidence were not significant in the path analysis.

## Discussion

Socio-ecological drivers of human malaria have been identified at global, national and subnational spatial scales [26–28], but it is currently unclear whether associations at large spatial scales hold at smaller spatial scales relevant for malaria control efforts by local authorities [14]. Here, we used the rural district of Ifanadiana, in south-eastern Madagascar, to investigate the relationship between hypothesized social and environmental determinants of malaria and malaria incidence at a very fine spatial scale. For this, we used a geographically-explicit dataset of malaria incidence at the Fokontany-level (a village or groups of villages) previously adjusted for known underreporting drivers in passive surveillance systems, and combined it with field surveys and remotely-sensed data via two separate statistical frameworks to account for direct and indirect pathways. We found that widely accepted drivers of malaria, including precipitation and temperature, were still relevant to predict incidence at fine spatial scales in our setting, in addition to identifying other land cover and socio-economic variables specific to the study region. Together, these factors allowed us to identify more than half of communities in the top quintile for malaria transmission for the 2016–2017 malaria season (compared to a 20% accuracy by chance), and explain over three quarters of the variation in malaria incidence rank.

Despite the very local scale of our study, large variations in temperature, both across space and time, occurred in Ifanadiana district. However, we only found an association between malaria incidence and temperature using a spatio-temporal GLM, and not in a spatial path analysis where data was averaged over time. This could be explained by the fact that, once averaged over time, mean land surface temperature only ranges between 22 and 28°C across Fokontany. In addition, malaria transmission is not only affected by mean temperature, but also by temperature variation, which could not be captured in the path analysis. Malaria dynamics have been shown to be strongly associated with temperature, which affects different stages of the mosquito larval development and mosquito lifespan [29,30]. It has been suggested that the effect of temperature on malaria is unimodal, with a predicted optimal temperature for malaria transmission of 25°C for *Anopheles* mosquito species and *Plasmodium falciparum* [22]. Previously, the association between temperature and malaria outcomes has been studied at relatively large scales, where heterogeneity can be larger [8,31–33]. Our analysis supported a unimodal relationship between temperature and malaria incidence. In addition, we found that malaria incidence was more likely to be non-zero when mean temperature was higher, suggesting that malaria transmission may be cold-limited in Ifanadiana district even during the warm, high transmission season.

The relationship between malaria and precipitation is complex and more context-specific than with temperature. Precipitation creates breeding grounds for mosquitoes but can also wash away larvae [34]. Therefore, depending on the local climate and mosquito life cycle stage, there may exist positive [35], negative, or unimodal relationships [28] between precipitation and malaria. We found evidence of a positive relationship between precipitation during the previous month and malaria incidence in our spatiotemporal model, supporting the ‘breeding grounds’ hypothesis. Unlike temperature, this relationship was also supported by our spatial SEM, where it had both a direct effect on malaria and an indirect effect via its association with larger areas of rice fields. This suggests that spatial variation in precipitation was large enough to affect malaria transmission patterns, by affecting the distribution of mosquito habitats such as rice fields and smaller pools.

Overall, land use emerged as an important ecological predictor of local malaria transmission in Ifanadiana. In this analysis, we considered three factors representing land cover at the Fokontany level: proportion of residential areas, proportion of rice fields and forest loss. We did not find any relationship between forest loss in the last three years and malaria incidence. Some studies have found a positive relationship between malaria and deforestation [36,37], with intact forests supporting non-vector species of mosquitoes while deforested areas provide vector habitat through agriculture, timber extraction and mining [38]. In south-eastern Madagascar, *Anopheles* mosquito species have been found to be rare in forested areas, while being very common in agricultural areas and near livestock pens [21]. Irrigated agriculture, in particular, has been identified as a strong predictor of *Anopheles* mosquito habitat across Madagascar [39], and the proportion of rice fields was positively associated with malaria in our spatiotemporal model as well. However, an increase in malaria with deforestation is characteristic of the frontier stage, when extensive changes in land use are occurring [38,40]. Instead, the majority of Fokontany in Ifanadiana are characterized by an open fragmented landscape and limited forested areas (Fig 1B), and would not be considered a frontier environment. Finally, Fokontany with a larger proportion of residential areas had lower malaria incidence. These results suggest that malaria transmission is associated with rural areas in the district, which have higher agriculture and irrigation relative to more urbanized areas (larger towns). This is consistent with previous findings, with lower malaria prevalence in children in urban areas than in rural areas, across the sub-Saharan region [41] and Madagascar [39,42].

Ifanadiana is heterogeneous with respect to wealth and access to health care. Despite using incidence data already adjusted for underreporting due to biases in geographical and financial access to care, we still found an association with both average household wealth and distance to primary health centers. Specifically, we found that wealthier fokontany had higher incidence rates and that fokontany more distant from a primary health center had lower incidence rates. In Ifanadiana, household wealth is concentrated along major transit axes, and health centers are located in larger towns but many populations live 2-6h away. Rather than reflecting social drivers of malaria incidence, these findings are likely the result of differential access to healthcare, a known phenomenon in Ifanadiana [43,44]. This reflects the fact that people who live further away from a health center and who have less economic resources are less likely to access health centers and be diagnosed with malaria [45,46].

Overall, the SEM allowed for a more nuanced understanding of local malaria transmission, revealing how socio-environmental factors affected malaria via direct and indirect pathways. For example, we found a negative relationship between the wealth index and forest loss. The relationship between poverty and deforestation is complex and may vary between regions and countries [47,48]. Our results suggest that poverty in Ifanadiana may drive people to depend on logging for income. The link between deforestation and poverty has been previously explored in other areas. For example, Jones et al. (2020) [49] found that clinic discounts were associated with a 70% reduction in deforestation in Borneo, as well as increased clinic usage. We also found higher reported bed net use was not associated with a change in malaria incidence in the spatiotemporal GLM, even though bed net use has generally been found to be an effective malaria prevention tool, including in Madagascar [45]. Results from the SEM suggest that bed net use was high in areas where climate was mediating a higher transmission risk (areas with higher temperature and precipitation), and when that was accounted for, it had a protective, although non-significant, effect on malaria spatial patterns.

While the primary purpose of this study was to investigate predictors of malaria at small spatial scales, we also explored the predictive potential of our GLM model. Despite a relatively high error (RMSE of 50 per thousand per month, compared to an average incidence of 61 per thousand per month during the high season), our model performed well when predicting the rank of each fokontany, both for within- and out-of-sample predictions. This difference in model performance can be explained by the large observed range in malaria incidence from 0 to 844 per thousand per month. The error associated with our model made it difficult to distinguish between Fokontany with small or intermediate levels of malaria incidence but allowed us to identify Fokontany with the highest malaria incidence. More specifically, we correctly identified 80% of the Fokontany above the median malaria prevalence, based on data from the two previous seasons. This would be of help to inform local intervention efforts from routine surveillance data, using relative rather than absolute metrics to identify Fokontany of concern. It is important to note that the time series used here are too short to allow modelling of longer-term temporal trends, so that predictions built from our 4-year period could be inadequate for future years due to anomalies, extreme weather events, or other factors not observed within our study period. The reliability of forward predictions could be increased as additional malaria and predictor data become available and longer time series are used. Longer time series would also allow for more explicit time series analysis. Finally, predictive power could also be increased in future work by increasing the number of predictors or by using specialized models that optimize predictive power at the expense of explanatory power, such as Support Vector Regression, Random Forest regression and other machine learning and deep learning models [50–52].

While we had access to health system, household survey and land cover data at high spatial resolution within one rural district, many of the limitations of our models were still due to either limited resolution in space or time or missing data. For example, the resolution of rainfall data was limited spatially while the resolution of detailed land use data was limited temporally. As a result, any directional relationship between malaria incidence and changes in land cover (other than deforestation) could not be explored. In addition, collinearity between some of these land cover variables decreased our ability to discern their respective impact. For this project, we used a dataset that adjusted from known biases in passive surveillance, which are difficult to correct completely [18,19]. If there were remaining biases due to factors other than those we identified and accounted for in our zero-inflated model (wealth, distance to healthcare), this could affect the spatiotemporal structure of malaria incidence and could impact our conclusions. We corrected for spatial autocorrelation using a Matern covariance structure but some spatial structure remained in the residuals, suggesting that other unknown variables correlated in space might contribute to malaria in this area. Finally, our study was undertaken in only one district of Madagascar, which limits its generalizability to other settings. Thus, methods developed here should be replicated elsewhere or expanded to larger areas in order to explore their wider applicability.

### Conclusion

This paper represents one of the first attempts to explain and predict malaria over fine spatial scales from routine passive surveillance data. We found that climatic, land use and socio-economic variables can be significant predictors of malaria incidence even at such fine scales, providing key insights for a context-specific understanding of local malaria transmission. In addition, our explanatory model performed well when predicting malaria dynamics, paving the way for more predictive frameworks that could help inform local health actors with the implementation of malaria control activities. Future work should focus on improving the spatiotemporal resolution of socio-environmental drivers of malaria and developing automated data pipelines and predictive frameworks to facilitate the integration of nowcasting or forecasting systems within local program implementation in areas of high malaria transmission.

## Materials and methods

### Study site

Ifanadiana is a rural district located in southeastern Madagascar, and is home to approximately 200,000 people across 195 Fokontany, the smallest administrative unit comprising a village or group of villages (average size of 20 km^2^ in Ifanadiana). The district is characterized by heterogeneous socio-economic, land use and climatic conditions, as well as malaria incidence (Fig 1A), which make it an ideal setting for this study. The dominant land class across the district is open or degraded forest (i.e. ‘savannah’), with the exception of Ranomafana National Park, a heavily forested protected area to the west of the district (Fig 1B). Rice fields and residential areas are interspersed throughout the district, among the savannah. The district is also characterized by a strong altitude gradient from east (100m) to west (1100m), which influences precipitation and temperature patterns (S1 Fig).

The passive malaria surveillance system in Ifanadiana relies on reported cases from primary health care facilities, each serving a population of about 10,000 people and an average catchment area of about 200 km^2^. The populations’ wealth and health care access are influenced by their proximity to the main paved road crossing the district and to health facilities, which are located in the larger towns (Figs 1A and S1) [53]. More than 75% of the population in Ifanadiana lives more than an hour away from a primary health care facility, more than one third lives over two hours away, and over one in ten people live further than three hours away [54]. Primary care utilization decreases exponentially with increasing distance and travel time to these health facilities [44]. In 2014, the NGO Pivot partnered with the Ministry of Public Health with the goal of strengthening the health system at all levels of care and ultimately ensuring universal access to health care, including malaria diagnosis and treatment. The intervention combines improving health system readiness (infrastructure, staffing, equipment, and medicine), integrated clinical programs, and robust data collection systems to inform program implementation. This work was done in the context of current efforts to improve malaria surveillance and control activities in the district.

### Data acquisition and processing

#### Malaria incidence data

We used a dataset of monthly malaria cases at the Fokontany level from January 2014 to December 2017, previously adjusted from passive surveillance data in Ifanadiana [18]. Methods used to produce this dataset are described in detail by Hyde et al. [18]. Briefly, de-identified patient-level data were collected from the 19 public health centers across Ifanadiana (covering 195 Fokontany), including age, Fokontany of residence, and malaria status, which was determined using rapid diagnostic tests. Use of Ministry of Health (MoH) data for this study was authorized by the Secretary General of the MoH, by the Medical Inspector of Ifanadiana district, and by Harvard’s Institutional Review board (IRB). The IHOPE cohort study was approved by the Madagascar National Ethics Committee and Harvard Medical School IRB. Population data was obtained from the Ministry of Public Health to calculate per-capita incidence estimates. Individual-level data was then aggregated to obtain monthly malaria incidence per capita at the level of the Fokontany for individuals of all ages, and for children under five years old. This dataset was finally adjusted for a variety of factors known to bias passive surveillance data, including distance to healthcare, existence of fee reimbursement systems and number of staff. For this, a benchmark multiplier was combined with a health care utilization index obtained from statistical models of non-malaria patients. Variations to the multiplier and several strategies for pooling neighboring communities together were explored to allow for fine-tuning of the final estimates. The resulting dataset was validated based on the reduction in said biases and through comparisons to areas with optimal health care access [18]. Here, we focus our analysis on the adjusted malaria incidence rates during the malaria high transmission season, which occurred from November to May (S1 Fig), to reduce the effect of missing data and false zeroes.

#### Climatic variables

We included two climatic variables known to influence malaria dynamics: temperature and precipitation [8,32,55]. Precipitation is an important driver of seasonal mosquito population dynamics and temperature determines larval mosquito and parasite development rates [30]. We obtained monthly land surface temperature (LST) by averaging biweekly data points from January 1^st^ 2014 to December 31^st^ 2017 from the thermal infrared band (Band 10) of Landsat 8 satellites [56], which has a spatial resolution of 30m (Table 1). We summed daily precipitation over each month to obtain monthly precipitation during the same period from the CHIRPS dataset, which provides rainfall estimates at a ~5km resolution from rain gauges and satellite data [57]. From these raster data and using Fokontany administrative limits, we calculated for each Fokontany total monthly precipitation, as well as spatial minimum, maximum and mean monthly LST. We excluded from the analysis anomalous LST (mean and minimum temperatures below 0°C and -5°C respectively) and capped maximum LST at 50°C. Temperature has been shown to have non-linear effects on malaria: transmission potential is a unimodal, symmetrical function of temperature with an optimum around 25°C and critical thermal thresholds below 17°C and above 34°C [22]. We thus defined a temperature suitability index by taking the squared difference between observed mean temperatures and this optimal temperature of 25°C.

**Table 1:** Predictor variables.

Predictor		Model	Spatial scale	Temporal scale	Source
Detection	Wealth score	GLM, SEM	Fokontany	Every two years (collected in 2014 2016, 2018)	[62]
	Distance to health center	GLM, SEM	Fokontany	Fixed (collected in 2018)	[54]
	Distance to road	SEM	Fokontany	Fixed	[54]
Behavioural	Bed net use (high season)	GLM, SEM	Fokontany	Every two years (collected in 2014, 2016, 2018)	[62]
Land use	Altitude	SEM	10x10m	Fixed	
	Residential area	GLM, SEM	10x10m	Fixed (collected in 2018)	[54]
	Rice field area	GLM, SEM			
	Distance from residential areas to forest	SEM			
	Forest edge	SEM			
Forest loss	Mean loss over last 3 years	GLM	30x30m	Yearly	Global Forest Change
	Mean loss over last 10 years	SEM			
Temperature (1-month and 2-month lag)	Min	GLM	30x30m (resampled)	Monthly (mean monthly value)	USGS Landsat 8, Band 10
	Max	GLM			
	Mean	GLM, SEM			
	Mean – suitability index	GLM			
Precipitation	Total	GLM, SEM	5566x5566 m	Monthly	CHIRPS

#### Land cover variables

We included predictors related to vegetation cover, land use and land use change which influence the distribution of mosquito breeding habitat, and may influence human behavior. We used qGIS [58] and ArcGIS [59] to calculate the proportion of each land cover class in each Fokontany from a land use map (Fig 1B) developed by Ihantamalala et al. [54]. This dataset was created via semi-supervised classification of Sentinel-2 imagery with identification of training data via OpenStreetMap, as described in Ihantamalala et al. [54]. Even though these land use features were obtained in 2018, they tend to be relatively stable over the short term in a rural area such as ours, so they were assumed to be a good representation of land cover for the period 2014–2017. Water bodies mostly included rivers and were not included in this analysis as moving water is not suitable mosquito breeding habitat. In addition, forest habitat was also excluded and replaced with other land cover metrics relevant to malaria transmission, such as distance from residential areas to forest, and forest edge [60]. This is because large forested areas are arbitrarily divided between different Fokontany, possibly biasing the analysis. These metrics were calculated using the original map in ArcGis.

We used data from the Global Forest Change dataset to determine the mean forest loss over the previous 3 and 10 years for each Fokontany and year during our study period [61]. The Global Forest Change dataset provides a global estimation of forest loss between 2000 and 2020 based on Landsat satellite data, including the year during which a pixel of 30m by 30m was deforested, if it was. We first used qGIS to visualize this information per year for Ifanadiana district and to calculate the proportion of deforested pixels for each year and for each Fokontany between 2004 and 2017. Finally, for each year during our study period (2014 to 2017), we estimated the average of that proportion over the last 3 and 10 years.

#### Socio-demographic variables

We included in our models important social and behavioral attributes of communities that can influence health-seeking behavior. Some of these (e.g., distance to health center) were already used in the initial adjustments of the malaria incidence dataset, but we included them here to account for residual uncorrected biases in malaria detection.

The distance of each Fokontany population to the nearest health center was obtained from Ihantamalala et al. [54]. Briefly, the shortest distance between each building in a Fokontany and the closest health center was calculated using the OSRM software, based on a full mapping of over 100,000 buildings and 23,000 km of footpaths previously conducted on OpenStreetMap [54]. Average distance to the nearest road was calculated by taking the average Euclidian distance to a road for every 100x100m pixel in the Fokontany.

We obtained household wealth and bed net use rates from a district-representative longitudinal cohort, which sampled 1600 households from 80 clusters across Ifanadiana every two years (2014, 2016 and 2018), selected through a two-stage cluster sampling scheme [62]. Data was collected by the Madagascar National Institute of Statistics via household and individual surveys based on the internationally validated Demographic and Health Surveys (DHS), which include information on different indicators of health and financial well-being. From this, we estimated a wealth index using standard DHS methods [63]. Briefly, a principal component analysis of household durable assets was estimated, which included access to electricity, water and toilets, material of roofing for houses, number of residents per bedroom and cooking fuel among others. Averages per cluster were estimated for each of the cohort variables each year, and these were extrapolated from clusters using inverse distance weights, obtaining a raster for the whole district. From this interpolated raster, we then averaged them over each Fokontany and year. Finally, wealth scores were interpolated to obtain monthly estimates from survey data obtained in 2014, 2016 and 2018 (Table 1).

#### Data processing

Data processing was performed in R. Residential area, rice field area, wealth score, distance between residential areas and forest, forest edge, distance to health center, forest loss and precipitation were log-transformed to correct for large deviations from normality. All variables were then scaled. Maps of all raw predictor variables are available in S2 Fig.

### Mixed-effect GLM

We fit a mixed-effect model with a zero-inflated negative binomial distribution to monthly malaria incidence at the Fokontany level using the glmmTMB package [64]. We used the AIC criterion to compare the fit of different distributions. In addition, we used the DHARMa package to perform residual diagnostics and confirm good model fit of the zero-inflated negative binomial model (KS test, p = 0.5) [65]. A zero-inflated negative binomial is used to represent count data that is over dispersed and has excess zeroes, which are assumed to be generated by two different processes. For example, zeroes in our dataset may be due to true absence of malaria incidence or to very low access to health care leading to underreporting. A more complete interpretation of the model is provided in the Results and Discussion section. We included Fokontany and month of the year as crossed random effects to account for repeated observations across time and space.

Fixed effects were determined using a two-step variable selection process, using AICc as our measure of fit. In both steps, five variables were always included based on the existing literature and exploratory analysis: bed net use, wealth score, distance to health center, residential area and rice field area. During the first step, we evaluated the model for all possible combinations of variables, listed in Table 1. In this step, we constrained models to always include all temperature and precipitation variables (either 1-month lag or 2-month lag), based on previous literature [8,22,32,55]. During the second step, we explored the influence of the three temperature variables by fixing all variables from the model with the lowest AICc in the first step and comparing models that included all possible combinations of minimum, mean, and maximum temperature. We present the model from the second step with the lowest AICc in the main manuscript. The steps described above are summarized in S2 Table. The model we present here was broadly consistent to the conditional average of the top 10% of all models explored (as determined by AICc) (S3 Table).

After variable selection, we assessed the spatial and temporal autocorrelation in the model’s residuals. We tested for temporal autocorrelation using a Durbin-Watson test on residuals aggregated over space. Spatial autocorrelation was assessed with a Moran’s I test on residuals aggregated over time and using the latitude and longitude of each Fokontany to make a distance matrix. We found both temporal (DW = 0.54, p < 0.0001) and spatial autocorrelation (I = 0.10, p < 0.0001) in the residuals of the model using month and Fokontany as random effects. We then updated our model structure to account for temporal autocorrelation by including a Ornstein–Uhlenbeck (OU) covariance structure, which allows for uneven time steps (only the high malaria season is used in this model). We fit a OU covariance matrix for each commune, which was sufficient to remove temporal autocorrelation in the residuals (DW = 1.8, p = 0.30). We also updated our model structure to use a Matern covariance structure using latitude and longitude to address spatial autocorrelation. The residuals of the resulting model showed reduced but still significant spatial autocorrelation (I = 0.23, p = 0.0002). We use the full model with OU and Matern covariance structure to obtain the model coefficients presented in this work and use the model with month and Fokontany as random effects for model predictions as to not rely on past malaria incidence for predictions and because the Matern covariance structure does not improve predictions. Coefficients for the model used for predictions are presented in S4 Table and equations for both model are available in S1 Text.

In our analysis, we first trained the model on the whole set of observations and then obtained in-sample predictions. For out-of-sample predictions, we trained the model on the 2014–2015 and 2015–2016 malaria seasons and tested it on the 2016–2017 season. Finally, we explored the contributions from different parts of our model (fixed vs. random, conditional vs. zero-inflated). We used the package ‘ggeffects’ to explore the marginal effects of our predictors [66]. We also fit the model with fixed effects only and compared its predictive performance to the full model.

### Structural equation modeling (SEM)

We used the piecewiseSEM package in R to explore putative relationships between our predictor variables in mediating malaria incidence, by accounting for indirect pathways and relationships between the explanatory variables [67]. We conducted the SEM analysis using data aggregated across time by taking the temporal mean for each Fokontany. The set of variables with a direct path to malaria incidence was chosen *a priori* based on the results of our GLMM exercise. Further variables were included to test specific hypotheses, namely:

- Wealth and average distance to health centers are both correlated with average distance to roads (Figs 1A and S1).
- Forest loss is higher in areas with lower wealth score [47].
- Forest loss is associated with an increase in forest edge [68]. We decided not to include forest area directly as forest area and forest loss may be related in complex ways (forest loss decreases forest area, but also can only occur if forest is still present).
- Areas with more rice fields have higher precipitation [69].
- Precipitation and temperature are influenced by altitude [70,71].
- Bed net use is correlated with known malaria risk factors, including higher local precipitation and temperature. We hypothesize that bed net use increases in high-risk areas, because of intervention policies and behaviour change following high malaria incidence [72].

We chose these hypotheses based on the variables available in our dataset and the causal relationship we could test considering their temporal resolution. For example, we could not test causal relationships between land cover classes and deforestation as land cover data were collected once in time. It is important to note that, although the SEM provides insights that are complementary to those from the GLM, it was not within the scope of these study to link these two models explicitly in a unified statistical framework.

## Supporting information

S1 Fig (A) Distribution of adjusted malaria incidence per Fokontany per month, averaged over four years. (B) Average precipitation and mean land surface temperature (LST) per month, averaged across Fokontany. Precipitation is shown with grey bars and mean LST is shown with a red line.
(PDF)

S2 Fig Maps of all unscaled predictor variables considered in the GLMM. The Fokontany boundary shapefile is available from OCHA (https://data.humdata.org/dataset/cod-ab-mdg) under a CC BY 4.0 License.
(PDF)

S3 Fig Distribution of observed and predicted malaria incidence across Fokontany and across time for the high malaria season (November-May). Outliers are omitted.
(PDF)

S1 Table Exponential of GLM coefficient estimates for model with spatial and temporal covariance structures. (DOCX)

S2 Table Model selection summary. Note that wealth score, distance to health center, bed net use, residential area and rice field area are always included. Variables that were included as a set are shown in boxes. Variables always included in the second step are indicated with a F.
(DOCX)

S3 Table Conditional average of the top 10% models (as determined by AICc). (DOCX)

S4 Table Exponential of GLMM coefficient estimates for model with spatial and temporal random effects. (DOCX)

S1 Text Model equations. (DOCX)
