# Supplementary figures and images for: Climatic, land-use and socio-economic factors can predict malaria dynamics at fine spatial scales relevant to local health actors: Evidence from rural Madagascar
Source: PLOS Glob Public Health. 2023 Feb 22;3(2):e0001607. doi: 10.1371/journal.pgph.0001607 (PMC10021226; doi:10.1371/journal.pgph.0001607)

**A**

Malaria incidence (per thousand)

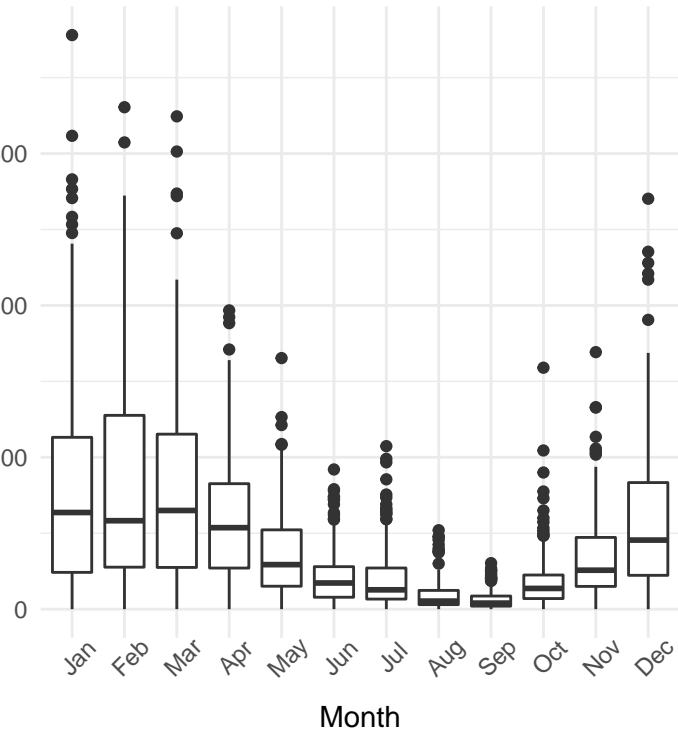**B**

Precipitation (mm)

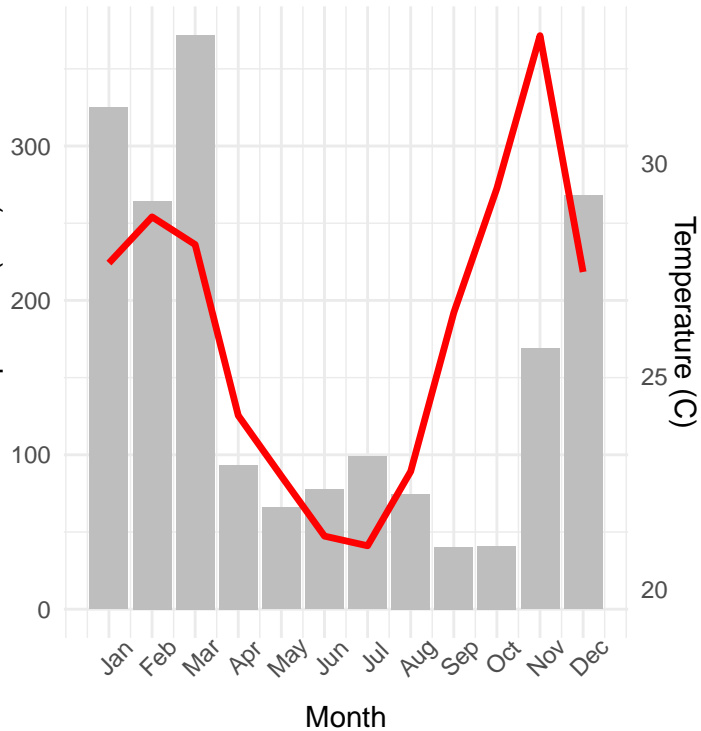

Supplement: S1 Fig — (A) Distribution of adjusted malaria incidence per Fokontany per month, averaged over four years. (B) Average precipitation and mean land surface temperature (LST) per month, averaged across Fokontany. Precipitation is shown with grey bars and mean LST is shown with a red line. (PDF) [file pgph.0001607.s001.pdf]

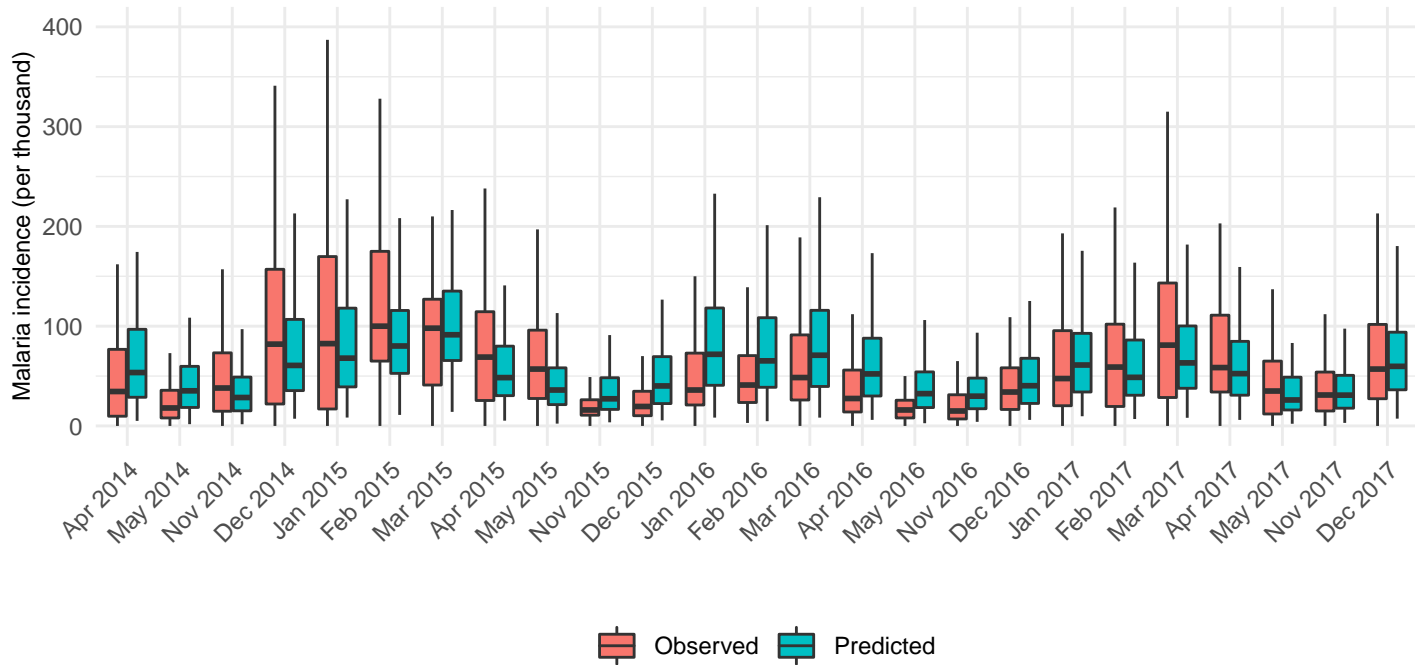

Supplement: S3 Fig — Outliers are omitted. (PDF) [file pgph.0001607.s003.pdf]
