# Supplementary material for: Climatic, land-use and socio-economic factors can predict malaria dynamics at fine spatial scales relevant to local health actors: Evidence from rural Madagascar
Source: PLOS Glob Public Health. 2023 Feb 22;3(2):e0001607. doi: 10.1371/journal.pgph.0001607 (PMC10021226; doi:10.1371/journal.pgph.0001607)
Supplement: S2 Fig — The Fokontany boundary shapefile is available from OCHA (https://data.humdata.org/dataset/cod-ab-mdg) under a CC BY 4.0 License. (PDF) [file pgph.0001607.s002.pdf]

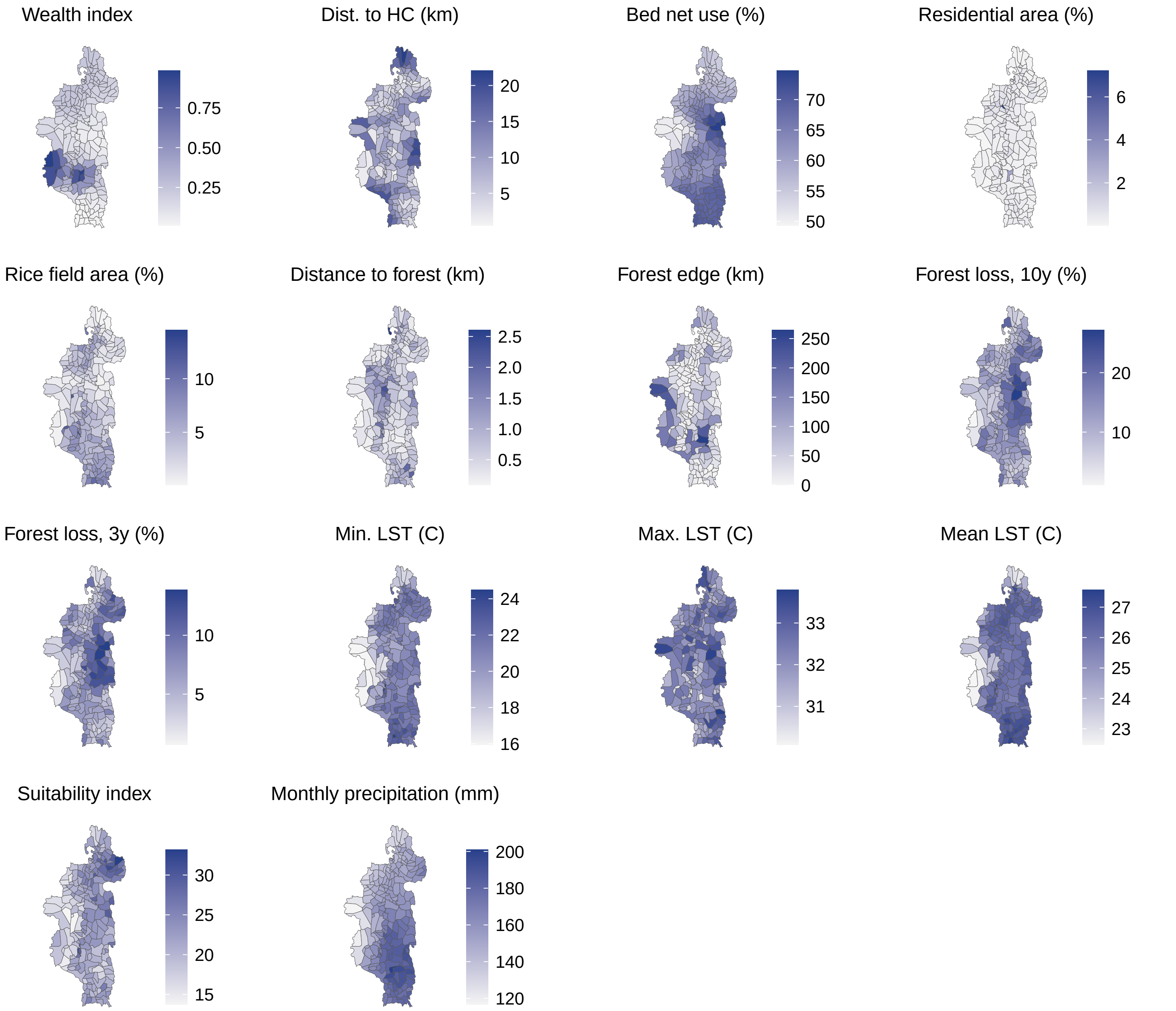
Wealth index
0.75
0.50
0.25
Dist. to HC (km)
20
15
10
5
Bed net use (%)
70
65
60
55
50
Residential area (%)
6
4
2
Rice field area (%)
10
5
Distance to forest (km)
2.5
2.0
1.5
1.0
0.5
Forest edge (km)
250
200
150
100
50
0
Forest loss, 10y (%)
20
10
Forest loss, 3y (%)
10
5
Min. LST (C)
24
22
20
18
16
Max. LST (C)
33
32
31
Mean LST (C)
27
26
25
24
23
Suitability index
30
25
20
15
Monthly precipitation (mm)
200
180
160
140
120
