# Supplementary material for: Climatic, land-use and socio-economic factors can predict malaria dynamics at fine spatial scales relevant to local health actors: Evidence from rural Madagascar
Source: PLOS Glob Public Health. 2023 Feb 22;3(2):e0001607. doi: 10.1371/journal.pgph.0001607 (PMC10021226; doi:10.1371/journal.pgph.0001607)
Supplement: S1 Table — (DOCX) [file pgph.0001607.s004.docx]

**S1 Table. Exponential of GLM coefficient estimates for model with spatial and temporal covariance structures.**

|  | **Conditional** | | **Zero-inflated** | |
| --- | --- | --- | --- | --- |
| **Variable** | **Estimate** | **CI (95%)** | **Estimate** | **CI (95%)** |
| Intercept | 32.3 | 26.81 - 38.89 | 0.00696 | 0 - 0.02 |
| Bed net use | 0.966 | 0.83 - 1.13 | 0.639 | 0.21 - 1.93 |
| Residential area (log10) | 0.858 | 0.78 - 0.94 | 1.56 | 0.89 - 2.74 |
| Rice field area (log10) | 1.12 | 1 - 1.26 | 0.734 | 0.44 - 1.23 |
| Distance to h.c. (log10) | 0.813 | 0.73 - 0.91 | 2.2 | 1.28 - 3.79 |
| Wealth score (log10) | 1.23 | 1.08 - 1.4 | 0.643 | 0.31 - 1.35 |
| Forest loss (log10) | 1.05 | 0.98 - 1.13 | 0.957 | 0.63 - 1.45 |
| Precipitation, 1-month lag (log10) | 1.33 | 1.26 - 1.41 | 0.64 | 0.45 - 0.91 |
| Mean LST, 1-month lag | 1.04 | 0.99 - 1.09 | 0.54 | 0.34 - 0.86 |
| Min LST, 1-month lag | 1.01 | 0.98 - 1.04 | 1.22 | 0.85 - 1.77 |
| Mean LST index, 1-month lag | 0.97 | 0.94 - 1 | 1.31 | 1.02 - 1.69 |
