# Supplementary material for: Climatic, land-use and socio-economic factors can predict malaria dynamics at fine spatial scales relevant to local health actors: Evidence from rural Madagascar
Source: PLOS Glob Public Health. 2023 Feb 22;3(2):e0001607. doi: 10.1371/journal.pgph.0001607 (PMC10021226; doi:10.1371/journal.pgph.0001607)
Supplement: S2 Table — Note that wealth score, distance to health center, bed net use, residential area and rice field area are always included. Variables that were included as a set are shown in boxes. Variables always included in the second step are indicated with a F. (DOCX) [file pgph.0001607.s005.docx]

**Table S2.** Model selection summary. Note that wealth score, distance to health center, bed net use, residential area and rice field area are always included. Variables that were included as a set are shown in boxes. Variables always included in the second step are indicated with a F.

| **Variables** | **Step 1** | | **Step 2** | |
| --- | --- | --- | --- | --- |
|  | Included | Selected | Included | Selected |
| Distance to forest | x |  |  |  |
| Forest edge | x |  |  |  |
| Forest loss (3 years) | x | x | F | F |
| Forest loss (10 years) | x |  |  |  |
| Min LST (1-month lag) | x | x | x | x |
| Max LST (1-month lag) | x | x | x |  |
| Mean LST (1-month lag) | x | x | x | x |
| Mean LST - SI (1-month lag) | x | x | x | x |
| Precipitation (1-month lag) | x | x | F | F |
| Min LST (2-month lag) | x |  |  |  |
| Max LST (2-month lag) | x |  |  |  |
| Mean LST (2-month lag) | x |  |  |  |
| Mean LST – SI (2-month lag) | x |  |  |  |
| Precipitation (2-month lag) | x |  |  |  |
