# Supplementary material for: Climatic, land-use and socio-economic factors can predict malaria dynamics at fine spatial scales relevant to local health actors: Evidence from rural Madagascar
Source: PLOS Glob Public Health. 2023 Feb 22;3(2):e0001607. doi: 10.1371/journal.pgph.0001607 (PMC10021226; doi:10.1371/journal.pgph.0001607)
Supplement: S3 Table — (DOCX) [file pgph.0001607.s006.docx]

| **Variable** | **Estimate** | **SE** | **p-value** |
| --- | --- | --- | --- |
| Intercept (cond.) | 3.85 | 0.107 | 0 |
| Bed net use (cond.) | 0.0158 | 0.0483 | 0.743 |
| Residential area (cond.) | -0.208 | 0.0526 | 7.61e-05 |
| Rice field area (cond.) | 0.212 | 0.0485 | 1.3e-05 |
| Distance to health center (cond.) | -0.278 | 0.0503 | 0 |
| Wealth score (cond.) | 0.175 | 0.0405 | 1.59e-05 |
| Forest loss, 3-year (cond.) | -0.0937 | 0.0215 | 1.24e-05 |
| Mean LST, one-month lag (cond.) | -0.119 | 0.0432 | 0.00596 |
| Min LST, one-month lag (cond.) | 0.0811 | 0.0176 | 3.9e-06 |
| Max LST, one-month lag (cond.) | 0.0756 | 0.0356 | 0.0334 |
| Mean LST index, one-month lag (cond.) | -0.0951 | 0.0131 | 0 |
| Precipitation, one-month lag (cond.) | 0.121 | 0.0386 | 0.00174 |
| Intercept (zi.) | -3.97 | 0.29 | 0 |
| Bed net use (zi.) | -0.861 | 0.223 | 0.000114 |
| Residential area (zi.) | 0.77 | 0.264 | 0.00349 |
| Rice field area (zi.) | -0.444 | 0.226 | 0.0495 |
| Distance to health center (zi.) | 0.836 | 0.237 | 0.000433 |
| Wealth score (zi.) | -0.41 | 0.196 | 0.0366 |
| Forest loss, 3-year (zi.) | -0.097 | 0.127 | 0.446 |
| Mean LST, one-month lag (zi.) | -0.343 | 0.305 | 0.261 |
| Min LST, one-month lag (zi.) | 0.244 | 0.147 | 0.0972 |
| Max LST, one-month lag (zi.) | -0.293 | 0.246 | 0.234 |
| Mean LST index, one-month lag (zi.) | 0.238 | 0.0832 | 0.00428 |
| Precipitation, one-month lag (zi.) | -0.443 | 0.181 | 0.0143 |
| Forest edge (cond.) | 0.054 | 0.0576 | 0.349 |
| Forest edge (zi.) | -0.0931 | 0.24 | 0.698 |
| Distance to forest (cond.) | 0.0354 | 0.0579 | 0.54 |
| Distance to forest (zi.) | -0.115 | 0.242 | 0.635 |
| Mean LST, two-month lag (cond.) | -0.104 | 0.051 | 0.0406 |
| Min LST, two-month lag (cond.) | 0.147 | 0.0179 | 0 |
| Max LST, two-month lag (cond.) | 0.0554 | 0.0368 | 0.133 |
| Mean LST index, two-month lag (cond.) | -0.0539 | 0.0186 | 0.00373 |
| Mean LST, two-month lag (zi.) | -0.392 | 0.489 | 0.422 |
| Min LST, two-month lag (zi.) | 0.377 | 0.152 | 0.0131 |
| Max LST, two-month lag (zi.) | 0.345 | 0.324 | 0.287 |
| Mean LST index, two-month lag (zi.) | 0.0442 | 0.151 | 0.77 |

**Table S3.** Conditional average of the top 10% models (as determined by AICc).
