# Supplementary material for: Climatic, land-use and socio-economic factors can predict malaria dynamics at fine spatial scales relevant to local health actors: Evidence from rural Madagascar
Source: PLOS Glob Public Health. 2023 Feb 22;3(2):e0001607. doi: 10.1371/journal.pgph.0001607 (PMC10021226; doi:10.1371/journal.pgph.0001607)
Supplement: S4 Table — (DOCX) [file pgph.0001607.s007.docx]

**S4 Table. Exponential of GLMM coefficient estimates for model with spatial and temporal random effects.**

|  | **Conditional** | | **Zero-inflated** | |
| --- | --- | --- | --- | --- |
| **Variable** | **Estimate** | **CI (95%)** | **Estimate** | **CI (95%)** |
| Intercept | 47.3 | 38.32 - 58.32 | 0.019 | 0.01 - 0.03 |
| Bed net use | 1.01 | 0.92 - 1.11 | 0.425 | 0.28 - 0.66 |
| Residential area (log10) | 0.805 | 0.73 - 0.89 | 2.23 | 1.35 - 3.68 |
| Rice field area (log10) | 1.23 | 1.12 - 1.35 | 0.657 | 0.42 - 1.02 |
| Distance to h.c. (log10) | 0.757 | 0.69 - 0.84 | 2.3 | 1.45 - 3.66 |
| Wealth score (log10) | 1.19 | 1.1 - 1.29 | 0.661 | 0.45 - 0.97 |
| Forest loss (log10) | 0.913 | 0.88 - 0.95 | 0.918 | 0.72 - 1.18 |
| Precipitation, 1-month lag (log10) | 1.12 | 1.04 - 1.21 | 0.644 | 0.45 - 0.93 |
| Mean LST, 1-month lag | 0.957 | 0.91 - 1.01 | 0.518 | 0.38 - 0.71 |
| Min LST, 1-month lag | 1.07 | 1.04 - 1.1 | 1.36 | 1.03 - 1.79 |
| Mean LST index, 1-month lag | 0.915 | 0.89 - 0.94 | 1.24 | 1.06 - 1.45 |
