# Supplementary material for: Climatic, land-use and socio-economic factors can predict malaria dynamics at fine spatial scales relevant to local health actors: Evidence from rural Madagascar
Source: PLOS Glob Public Health. 2023 Feb 22;3(2):e0001607. doi: 10.1371/journal.pgph.0001607 (PMC10021226; doi:10.1371/journal.pgph.0001607)
Supplement: S1 Text — (DOCX) [file pgph.0001607.s008.docx]

**S1 Text. Model equations**

1. **GLMM**

We use a zero-inflated negative binomial distribution, which assumes that the expected malaria count is a combination of a logit model and a count process. Zeroes can be attributed to either processes. The expected malaria count $\boldsymbol{y}$ in a Fokontany over a month is determined by a number $\boldsymbol{n}$ of variables $\boldsymbol{x}$(presented in Table 1) and their variable coefficients $\boldsymbol{\beta}$:

$$\boldsymbol{log}\left( \boldsymbol{y} \right)\boldsymbol{=}\sum_{\boldsymbol{i=1}}^{\boldsymbol{n}} \boldsymbol{\beta}_{\boldsymbol{i}}\boldsymbol{x}_{\boldsymbol{i}}\boldsymbol{+}\boldsymbol{\beta}_{\boldsymbol{0}}$$

For the model with random intercepts, we include a random intercept for each Fokontany $\boldsymbol{j}$ and month $\boldsymbol{k}$:

$$\boldsymbol{log}\left( \boldsymbol{y}_{\boldsymbol{j,k}} \right)\boldsymbol{=}\sum_{\boldsymbol{i=1}}^{\boldsymbol{n}} \boldsymbol{\beta}_{\boldsymbol{i}}\boldsymbol{x}_{\boldsymbol{i,j,k}}\boldsymbol{+}\boldsymbol{\beta}_{\boldsymbol{0,j}}\boldsymbol{+}\boldsymbol{\beta}_{\boldsymbol{0,k}}$$

For the model with a Matern covariance structure, the random intercept $\boldsymbol{\beta}_{\boldsymbol{0,j}}$ is spatially correlated, using a Matern covariance function (1). The Ornstein–Uhlenbeck temporal autocorrelation term behaves similarly to a AR1 process, while allowing for uneven time steps (2), and is determined for each commune. Coefficients are determined with the package glmmTMB (3).

1. **SEM**

We use the package piecewiseSEM (4) to specify our SEM. We only use linear regressions to describe the relationships between our variables:

$$\boldsymbol{y}\boldsymbol{=}\sum_{\boldsymbol{i=1}}^{\boldsymbol{n}} \boldsymbol{\beta}_{\boldsymbol{i}}\boldsymbol{x}_{\boldsymbol{i}}\boldsymbol{+}\boldsymbol{\beta}_{\boldsymbol{0}}$$

**References**

1. Matérn B. Spatial variation. Vol. 36. Springer Science & Business Media; 2013.

2. Uhlenbeck GE, Ornstein LS. On the theory of the Brownian motion. Phys Rev. 1930;36(5):823.

3. Brooks ME, Kristensen K, van Benthem KJ, Magnusson A, Berg CW, Nielsen A, et al. {glmmTMB} Balances Speed and Flexibility Among Packages for Zero-inflated Generalized Linear Mixed Modeling. R J [Internet]. 2017;9(2):378–400. Available from: https://journal.r-project.org/archive/2017/RJ-2017-066/index.html

4. Lefcheck JS. piecewiseSEM: Piecewise structural equation modeling in R for ecology, evolution, and systematics. Methods Ecol Evol [Internet]. 2016;7(5):573–9. Available from: http://dx.doi.org/10.1111/2041-210X.12512
